# Inhibition of miR-224-5p promotes osteogenesis in dental pulp stem cells by targeting the PTEN/PI3K/AKT axis

**DOI:** 10.1186/s13018-026-06853-w

**Published:** 2026-04-11

**Authors:** Zhihong Ke, Fan Yang, Ningkai Huang, Hongbing Lv

**Affiliations:** 1 Stomatological Hospital of Xiamen Medical College, No.1309, Lvling Road, Huli District, Xiamen, 361008 Fujian China; 2 Xiamen Key Laboratory of Stomatological Disease Diagnosis and Treatment, Xiamen, 361008 Fujian China; 3 Fujian Key Laboratory of Oral Diseases & Fujian Provincial Engineering Research Center of Oral Biomaterial & Stomatological Key Laboratory of Fujian College and University, School and Hospital of Stomatology, Fujian Medical University, Fuzhou, 350000 Fujian China; 4 Institute of Stomatology & Research Center of Dental and Craniofacial Implants, School and Hospital of Stomatology, Fujian Medical University, Fuzhou, 350000 Fujian China

**Keywords:** Human dental pulp stem cells, Tissue engineering, PTEN, PI3K/AKT signaling pathway, Osteogenesis

## Abstract

**Background:**

MiR-224-5p has been proven to play an important role in regulating cell differentiation. This study aimed to clarify the regulatory role and mechanism of miR-224-5p in the osteogenic differentiation of human dental pulp stem cells (hDPSCs), thereby laying a theoretical foundation for subsequent jaw defect repair.

**Methods:**

Human dental pulp stem cells (hDPSCs) were isolated, cultured, and sorted from healthy dental pulp tissues. We performed integrated bioinformatics analysis to screen and identify the potential targets and pathways of miR-224-5p involved in the osteogenic induction of hDPSCs. Subsequently, in vitro experiments were conducted. Plasmid transfection was used to regulate the overexpression and knockdown of miR-224-5p in hDPSCs, and the expression of osteogenesis-related proteins was detected. Furthermore, luciferase reporter assays and Western blot assays were used to confirm the direct targets of miR-224-5p, and rescue experiments were performed to verify the underlying mechanism.

**Results:**

The results demonstrated that overexpression of miR-224-5p inhibited the osteogenic differentiation of DPSCs, as reflected by the significantly decreased expression of osteogenic markers (OCN, Runx2, and ALP). In contrast, inhibition of miR-224-5p promoted the osteogenic differentiation of DPSCs. Bioinformatics analysis and dual-luciferase reporter gene assays indicated that miR-224-5p specifically targets the 3’ untranslated region of the PTEN gene. Rescue experiments further confirmed that miR-224-5p regulates this process by modulating the PTEN/PI3K/AKT pathway.

**Conclusions:**

Inhibition of miR-224-5p promotes osteogenesis in DPSCs by targeting the PTEN/PI3K/AKT signaling axis. These findings provide reliable evidence for the fabrication of three-dimensional tissue-engineered structures and further repair of maxillofacial bone defects.

**Supplementary Information:**

The online version contains supplementary material available at 10.1186/s13018-026-06853-w.

## Introduction

Oromaxillofacial bone defects commonly arise from trauma, tumor resection, and age-related degenerative changes. Once these defects develop, they can pose severe detriments to patients’ physiological functions and mental health [1]. Currently, the main treatment strategies for bone defect repair include bone transplantation and implantation of bioactive compounds [2, 3]. Nonetheless, natural bone grafts, including autografts, allografts and xenografts, have notable limitations such as delayed healing, donor shortage, infection risks and related postoperative morbidity [4, 5]. How to utilize the body’s own cells to promote osteogenesis at the defect site remains an unresolved clinical problem at present.

Non-coding RNAs (ncRNAs) act as pivotal regulators in the pathogenesis and progression of musculoskeletal disorders. Mounting evidence has uncovered their extensive potential for elucidating the molecular mechanisms underlying diverse pathological conditions, such as osteoporosis (OP), tendon homeostasis, and rheumatoid arthritis (RA) [6–9]. Additionally, long non-coding RNAs (lncRNAs) including CRNDE and HCG18 have been demonstrated to regulate fracture healing and the pathological progression of spinal tuberculosis through miRNA sponging mechanisms [10, 11]. MicroRNAs (miRNAs) represent a class of endogenous small noncoding RNAs that bind to the 3’-untranslated regions (3’-UTRs) of cognate target genes to suppress their translation, thereby modulating a spectrum of core biological processes, including cell proliferation, apoptosis, and differentiation [12, 13]. For instance, miR-217 has been verified to regulate the OPG gene and improve bone metabolic indices, offering a promising therapeutic target for postmenopausal osteoporosis [14]. MiR-204-5p attenuates the degree of intervertebral disc degeneration and preserves intervertebral disc height, thereby providing a novel therapeutic strategy for intervertebral disc degeneration [15]. Human dental pulp stem cells (hDPSCs) are a type of stem cell that share characteristics with mesenchymal stem cells (MSCs) and can be isolated from dental pulp tissue [16]. Compared with stem cells from the apical papilla (SCAPs)—a cell type widely investigated in dental pulp regeneration—DPSCs possess more accessible sources and simpler acquisition procedures [17]. We therefore sought to explore whether the distinct biological characteristics of these two stem cell populations are linked to disparities in their miRNA expression profiles. In our preliminary experiments, miRNA microarray profiling was performed in DPSCs and SCAPs, and miR-224-5p was screened out as the most markedly differentially expressed miRNA between the two cell types. Our follow-up studies further demonstrated that knockdown of miR-224-5p facilitates the proliferation and migration of DPSCs [18]. Through bioinformatics analysis, we preliminarily identified that the function of miR-224 in DPSCs may be associated with PI3K/AKT signaling, which is in line with the findings of other studies [19, 20]. In addition, numerous studies have validated that the PTEN/PI3K/AKT signaling axis is involved in regulating osteogenic differentiation [21, 22]. From a clinical perspective, it is imperative to induce DPSCs to regenerate clinically requisite tissues (e.g., dental pulp tissue and bone tissue) and engineer single cells into three-dimensional tissue constructs [23–25].

Therefore, our goal was to explore the effect of miRNA-224-5p expression on the osteogenic differentiation of hDPSCs and to clarify the underlying mechanism to provide data to support future tissue engineering research. In this study, we demonstrated that miR-224-5p promotes osteogenesis of human dental pulp stem cells (hDPSCs) in vitro. Mechanistically, miR-224-5p mediates osteogenic differentiation of hDPSCs via regulating the PTEN/PI3K/Akt signaling pathway.

## Methods

### Cell isolation and culture

All experiments were approved by the Ethics Committee of the Affiliated Stomatology Hospital, Fujian Medical University (Approval No. 2025ECSS094). Dental pulp tissues were extracted under sterile conditions from healthy impacted third molars or premolars extracted for orthodontic purposes in patients aged 13 to 23 years. The tissues were washed three times with sterile phosphate-buffered saline (PBS; HyClone, USA) supplemented with antibiotics (100 U/ml penicillin and 100 U/ml streptomycin; HyClone, USA). Subsequently, the pulp tissues were minced into small fragments and digested with 3 mg/ml type I collagenase (Gibco, USA) and 4 mg/ml dispase II (Sigma, USA) at 37 °C for 30 min. An equal volume of Dulbecco’s modified Eagle’s medium (DMEM; HyClone, USA) containing 15% fetal bovine serum (FBS; HyClone, USA) was then added to terminate the digestion. The mixture was then centrifuged at 1000 rpm for 5 min using a low-speed centrifuge. After the supernatant was completely discarded, the cell pellet was resuspended and evenly seeded into a 6-cm diameter culture dishes. Afterward, 2 mL of DMEM supplemented with 20% FBS and 1% penicillin‒streptomycin (P/S) was gently added to the dishes, and all the cultures were incubated at 37 °C in 5% CO_2_ for 7–10 days until cell confluence reached 70–80%, at which point trypsin (Gibco, USA) was used to digest the cells for passaging; these cells were considered the first passage. Cells at passages 2–3 were used for this study.

### Cell sorting, identification, and differentiation

Cell sorting was performed according to the manufacturer’s protocol for an immunomagnetic bead-based system (Miltenyi Biotec, Germany). Briefly, 1 × 10^7^ dental pulp cells (passage 3) were resuspended in 100 µL of cold (4 °C) reaction buffer, containing of 60 µL MACS Running Buffer, 20 µL of FcR Blocking Reagent, and 20 µL of Anti-Human STRO-1 MicroBeads (R&D Systems, USA). The suspension was incubated at 4 °C for 15 min in the dark, followed by magnetic separation using an MS column (Miltenyi Biotec, Germany) pre-equilibrated with buffer. STRO-1(+) cells retained on the column were eluted with 1 mL of buffer for expansion culture, and unlabeled cells were discarded. All steps were performed under sterile conditions.

### Flow cytometric analysis

Passage 3 STRO-1(+) cells were enzymatically digested, collected by centrifugation, and resuspended in sterile PBS. Following a second centrifugation step, the cell pellet was resuspended in Flow Cytometry Staining Buffer (FACS buffer; BD Biosciences, USA) to a final density of 1.0 × 10^6^ cells/mL. The cell suspension was then incubated with fluorochrome-conjugated monoclonal antibodies against CD34, CD45, CD73, and CD90 (HUABIO, China) in FACS buffer at 4 °C for 30 min in the dark. After incubation, the cells were washed three times with cold PBS to remove unbound antibodies. The final cell pellet was resuspended in 500 µL of PBS and immediately analyzed using a flow cytometer (BD, USA). At least 10,000 events per sample were acquired, and the data were processed using Tree Star FlowJo 10.10.0 software. Isotype-matched controls were applied to determine the extent of background staining, and results are reported as the percentage of marker-positive cells.

### Osteogenic and adipogenic induction

The multipotent differentiation potential of STRO-1(+) cells was evaluated using osteogenic induction medium (DMEM supplemented with 10% FBS, 10 nmol/L dexamethasone, 10 mmol/L β-glycerophosphate and 50 µg/mL vitamin C; Sigma, USA) and adipogenic induction medium (HUXXC-90031, OriCell, China). At predefined time points, the samples were stained with alkaline phosphatase, alizarin red, and oil red O solution (OILR-10001, OriCell, China).

### Dental pulp stem cells transfection

HDPSCs were seeded in 12-well culture plates and transfected when the cell confluency reached 70–80%. Before transfection, the culture medium was replaced with serum-free medium, and miR-224 mimics, miR-224 inhibitors, as well as their corresponding controls (Sangon Biotech, China) were introduced into the cells using Lipofectamine 2000 (Thermo Fisher Scientific, USA) transfection reagent. The corresponding sequences are illustrated in Table 1. After 6 h of transfection, the medium was replaced with complete growth medium, and the cells were cultured for another 48–72 h. The transfection efficiency was then evaluated, and the transfected cells were harvested for subsequent experiments.

**Table 1:** Sequences of the transfected plasmids

miRNA	Sequence
miR-224-5p mimic	5′-UCAAGUCACUAGUGGUUCCGUUUAG-3′
miR-224-5p inhibitor	5′-CUAAACGGAACCACUAGUGACUUGA-3′
mimic-NC	5′-UUGUACUACACAAAAGUACUG-3′
inhibitor-NC	5′-CAGUACUUUUGUGUAGUACAA-3′

### Quantitative real-time polymerase chain reaction (RT-qPCR)

Total RNA was extracted using TRIzol reagent (Ambion, USA), and complementary DNA (cDNA) was synthesized via reverse transcription using a HiFiScript cDNA First Strand Synthesis Kit (Vazyme, China) in accordance with the manufacturer’s protocol. The resulting cDNA was directly used for quantitative PCR (qPCR). For miRNA reverse transcription, miRNAs were subjected to polyadenylation prior to cDNA synthesis. qPCR was performed following the standard protocol of the SYBR Green Kit (Vazyme, China). The primer sequences are listed in Table 2.

**Table 2:** Sequences of primers used for qRT-PCR

Gene		Primer
Hsa-miR-224-5p	Forward	5′-CCGCTCAAGTCACTAGTGGTTCC-3’
	Reverse	5’-AGTGCAGGGTCCGAGGTATT-3’
PTEN	Forward	5’-CACCACTCACTACCACACCTA-3’
	Reverse	5’-TGACGAAGTGCCATAGTAGAGAT-3’
RUNX2	Forward	5’-CACCACTCACTACCACACCTA-3’
	Reverse	5’-TGACGAAGTGCCATAGTAGAGAT-3’
OCN	Forward	5’-CACACTCCTCGCCCTATTG-3’
	Reverse	5’-GGTCTCTTCACTACCTCGCT-3’
ALP	Forward	5’-CTGGACCTCGTTGACACCTG-3’
	Reverse	5’-TCCGTCACGTTGTTCCTGTT-3’
GAPDH	Forward	5’-GGTGTGAACCATGAGAAGTATGA-3’
	Reverse	5’-GAGTCCTTCCACGATACCAAAG-3’

### Alkaline phosphatase and alizarin red staining

Osteogenic differentiation was induced in the transfected dental pulp stem cells. Alkaline phosphatase (ALP) activity was quantitatively analyzed on Day 7, while alizarin red S (ARS) staining was performed for the quantification of mineralized nodules on Day 14. Cells were stained using an ALP detection kit (Beyotime, China) and an ARS detection kit (Solarbio, China) according to the manufacturers’ protocols. ALP activity was detected using an ALP detection kit. ALP activity was calculated according to the formula: ALP activity = absorbance value of the assay tube/total protein content (g).

### Dual-luciferase reporter gene and Western blotting assays

A wild-type 3′-untranslated region (3′-UTR) of PTEN containing a putative miR-224-5p binding site, together with a corresponding mutant 3′-UTR with a disrupted binding site, was constructed. All luciferase reporter assays were performed in 293T cells. In accordance with the manufacturer’s instructions for the Luciferase Assay System (Vazyme, China), the transfected cells were lysed 48 h after transfection, after which luciferase activity was measured.

Cells were lysed on ice using RIPA lysis buffer (Beyotime, China) supplemented with 1% phenylmethylsulfonyl fluoride (PMSF). Protein concentration was quantified with a BCA protein assay kit. Protein samples were mixed with 5× sodium dodecyl sulfate (SDS) loading buffer at a 4:1 ratio, boiled for 10 min to denature proteins, separated via precast SDS-PAGE, and transferred onto polyvinylidene fluoride (PVDF) membranes. The membranes were blocked with 5% bovine serum albumin (BSA) solution at room temperature for 1 h, then incubated with specific primary antibodies at 4 °C overnight. After washing with blocking buffer, the membranes were incubated with horseradish peroxidase (HRP)-conjugated secondary antibodies (HuaBio, China) at room temperature for 1 h. Finally, enhanced chemiluminescence (ECL) reagent was added in the dark for band development and exposure. Glyceraldehyde-3-phosphate dehydrogenase (GAPDH) was used as the internal reference to quantify the expression levels of target proteins, including RUNX2, ALP and OCN. Western blot images were visualized using Image Lab 3.0 software, and quantitative analysis was performed via ImageJ-win64.

### Statistical analysis

All experiments were performed in triplicate at minimum. Data are presented as means ± standard deviations (SDs). Statistical analyses were conducted using GraphPad Prism 9.0 software. Comparisons between two groups were analyzed via unpaired Student’s t-tests, and one-way analysis of variance (ANOVA) was applied for comparisons among multiple groups. A p-value < 0.05 was regarded as statistically significant.

## Results

### Identification of dental pulp stem cells

**Fig. 1:**
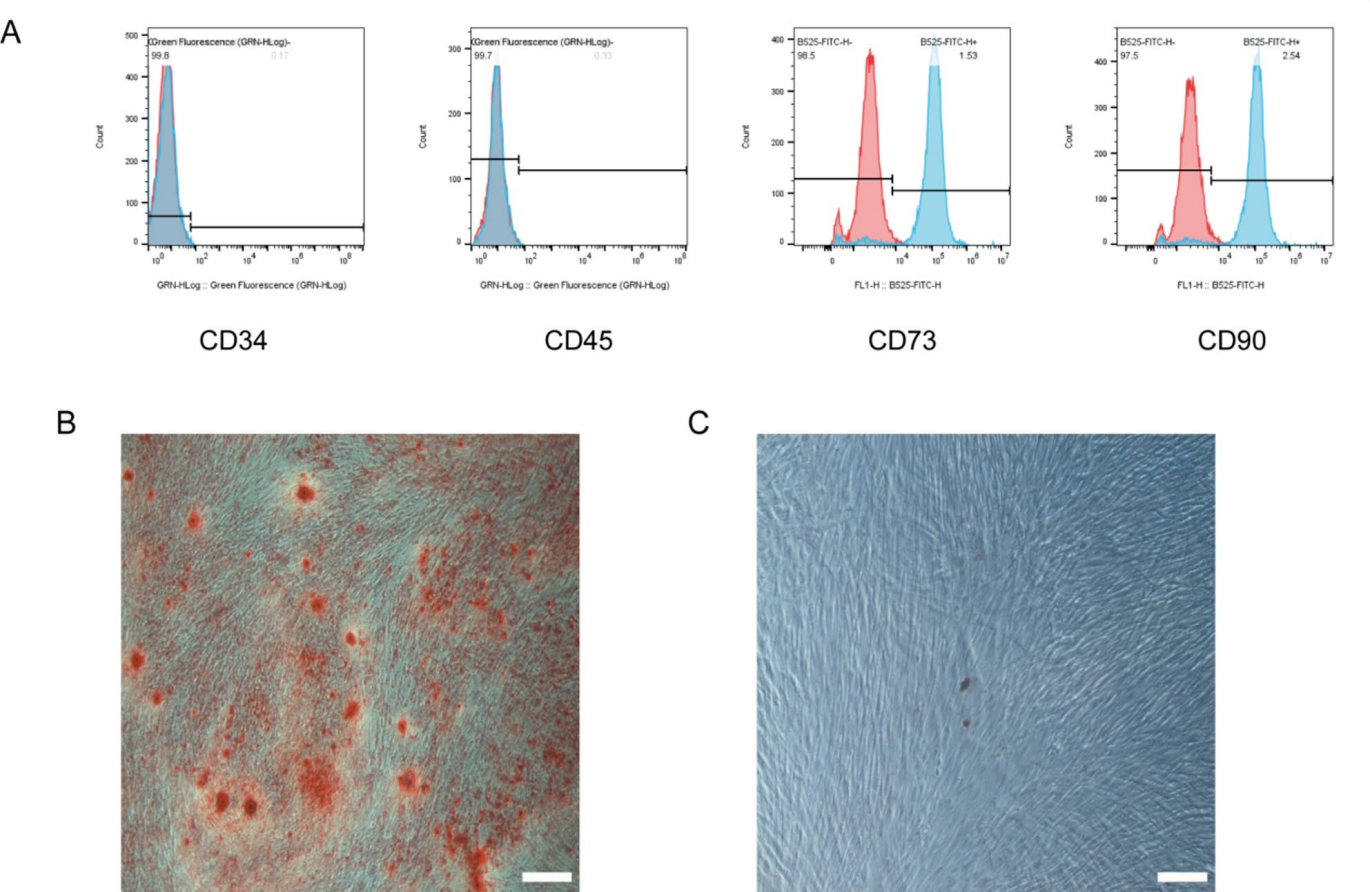
Surface markers of hDPSCs, including CD34(−), CD45(-)CD73(+), and CD90(+). Alizarin red staining, scale bar, 10×, 100 μm; and oil red O staining, scale bar, 10×, 100 μm

To investigate the characteristics of STRO-1(+) cells, we determined the expression of cell surface markers. Flow cytometric analysis revealed positive expression of the surface markers CD73 and CD90 and negative expression of CD34 and CD45 (Fig. 1A). Multidirectional differentiation induction showed that the cells could successfully be differentiated into osteoblasts (Fig. 1B) and adipocytes (Fig. 1C). Human dental pulp stem cells were successfully isolated [26].

### Transfection efficiency of plasmid expressing miR-224-5p

**Fig. 2:**
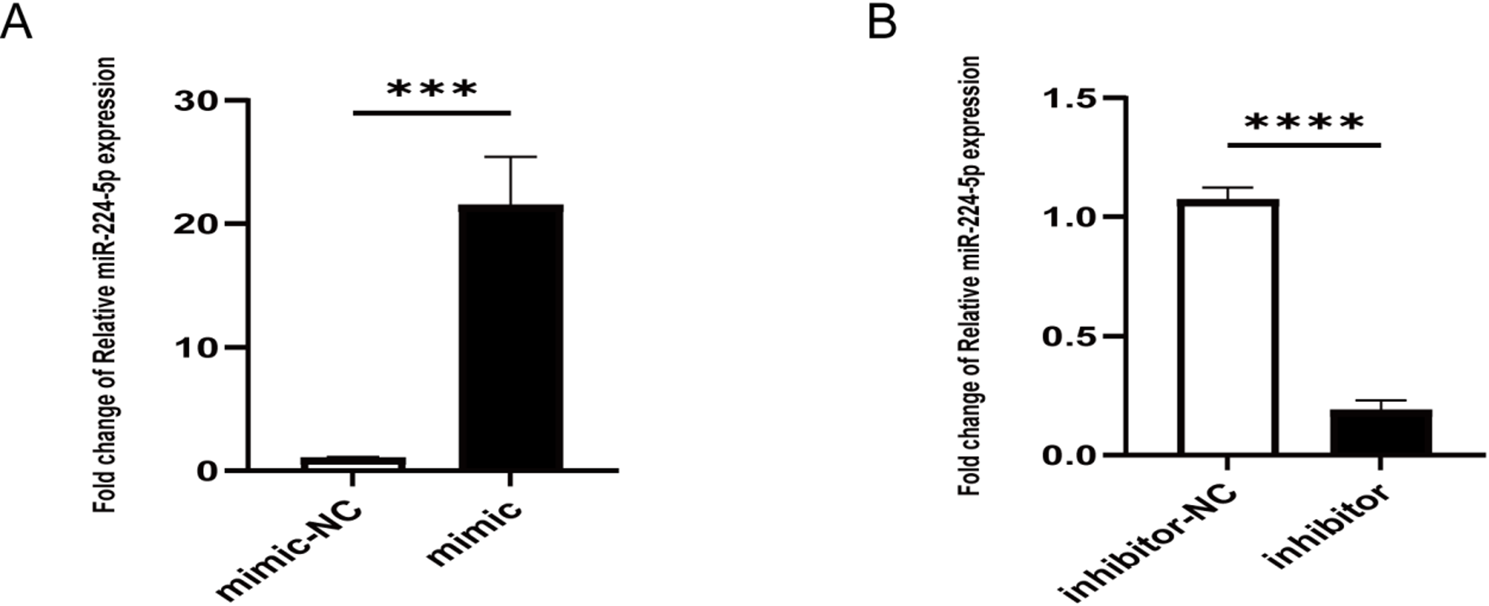
HDPSCs were transfected with either miR‑224-5p inhibitor or miR-224-5p mimic, or their controls. MRNA expression levels of **A** miR‑224-5p mimic and **B** miR-224-5p inhibitor in each group were detected. *** *p* < 0.001,**** *p* < 0.0001 compared with control

qRT‒PCR analysis confirmed that the expression level of miR-224-5p was significantly altered after transfection (Fig. 2). The expression of miR-224-5p differed greatly across the groups. Compared with the mimic negative control (NC) group, the expression level of miR‑224‑5p was significantly upregulated in the miR‑224‑5p mimic group (*p* < 0.001). In contrast, transfection with the miR‑224‑5p inhibitor led to a markedly decreased expression level of miR‑224‑5p relative to the inhibitor NC group (*p* < 0.0001).

### Effects of miR-224-5p overexpression/inhibition on the osteogenic differentiation of hDPSCs

**Fig. 3:**
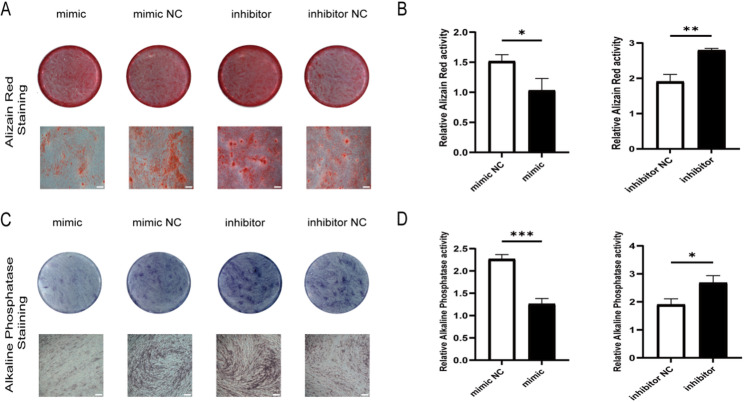
Formation of calcified nodules in hDPSCs subjected to miR-224-5p overexpression or inhibition and subsequently cultured in mineralization induction medium for 14 d. Scale bar, 10x, 100 μm (**A**). ALP staining results for DPSCs subjected to miR-224-5p overexpression or inhibition following mineralization induction for 7 d (**C**). Scale bar, 10×, 100 μm. The results of the quantitative analysis are shown in this figure **B** and **D**. * *P* < 0.05, ** *p* < 0.01, *** *p* < 0.001 compared with the control

Alizarin Red S staining revealed a significant increase in mineralized nodule formation in the miR-224-5p inhibition group, whereas mineralization was markedly inhibited in the overexpression group (Fig. 3A). The ALP staining results revealed the same trend (Fig. 3C). The results of the corresponding quantitative analysis are shown in Fig. 3B and D.

**Fig. 4:**
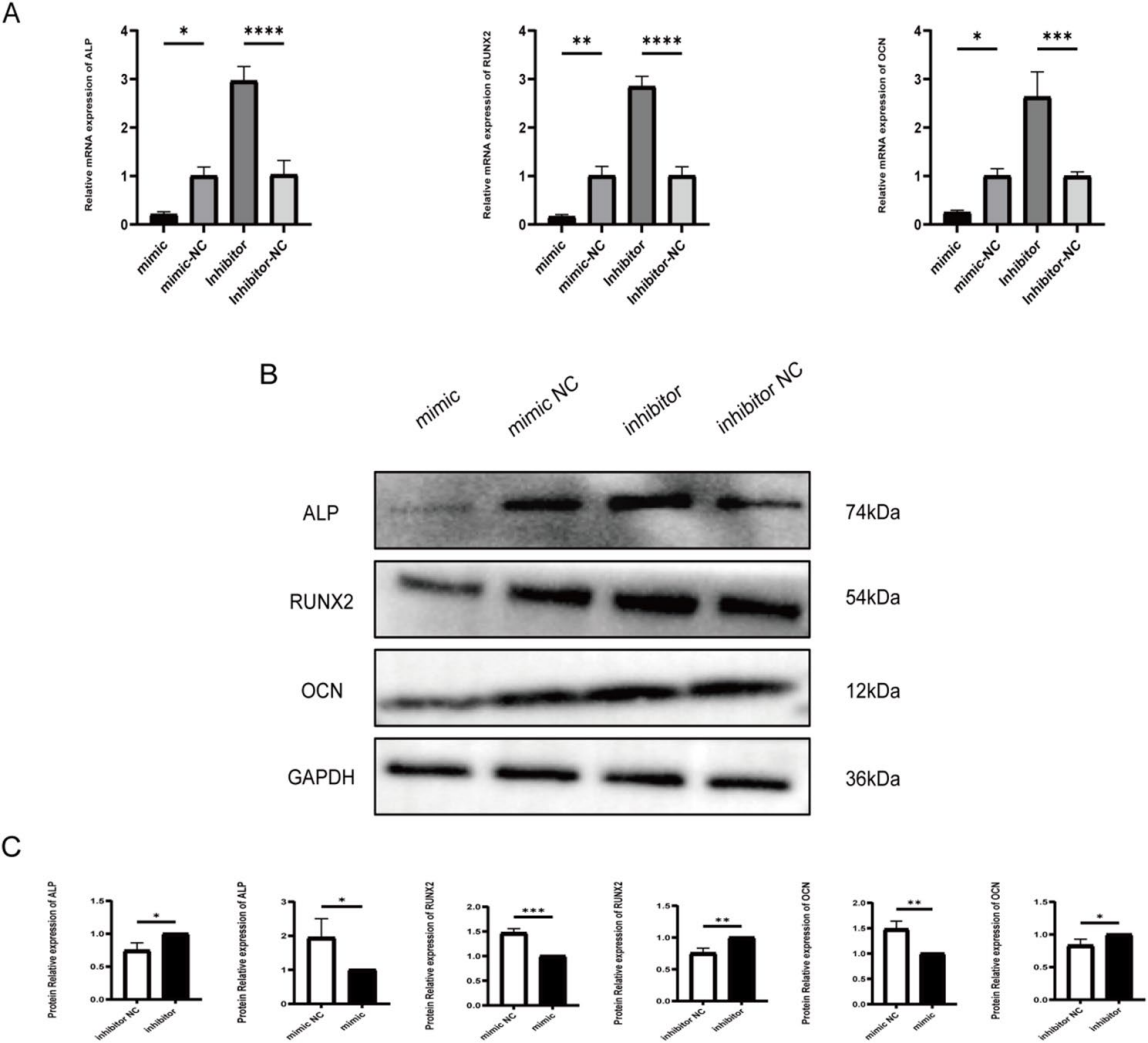
mRNA expression (**A**) and protein expression (**B**,** C**) of osteogenesis-related proteins ALP, RUNX2, and OCN. *P < 0.05, ** *p* < 0.01, *** *p* < 0.001, **** *p* < 0.0001 compared with the control

Furthermore, qRT‒PCR analysis of the expression levels of key osteogenic transcription factors (ALP, RUNX2, and OCN) showed that the mRNA levels of these genes were significantly up-regulated in the miR-224-5p inhibition group, but were markedly down-regulated in the overexpression group(Fig. 4A). Western blot analysis confirmed that the expression changes of osteogenesis-related proteins (ALP, RUNX2, and OCN) were consistent with the transcriptional trends (Fig. 4B). Quantitative analysis further revealed that inhibition of miR‑224‑5p significantly increased the expression of osteogenesis-related proteins, whereas overexpression of miR‑224‑5p markedly reduced their expression (Fig. 4C).

### PTEN was identified as a target gene of miR-224-5p

**Fig. 5:**
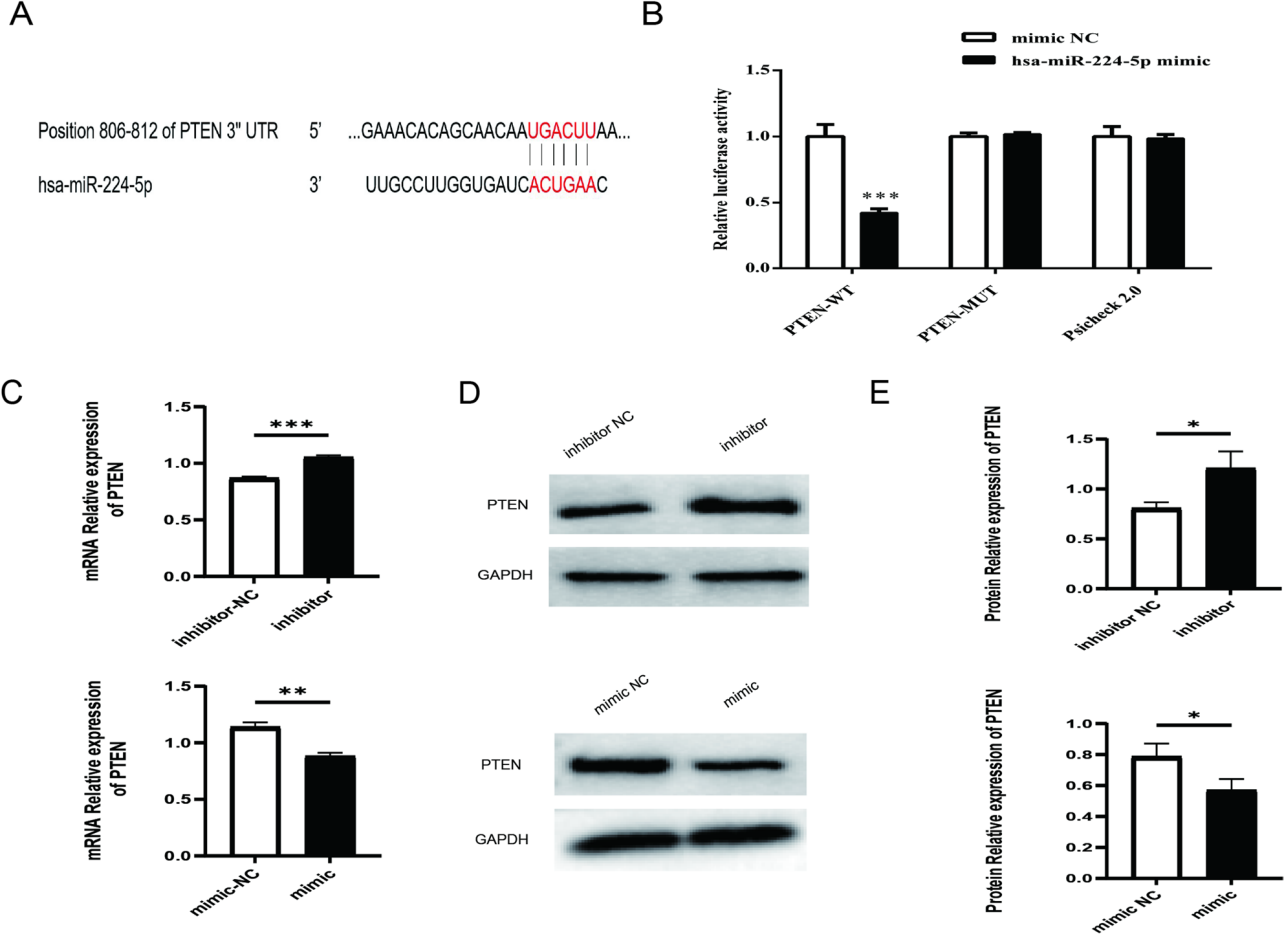
Bioinformatics analysis of miR‑224-5p and the 3’‑UTR of the PTEN gene (**A**). The results of the dual luciferase reporter assay revealed the targeting regulatory relationship between miR-224-5p and PTEN (**B**). Effect of miR-224-5p overexpression/underexpression on PTEN mRNA (**C**) and protein (**D**,** E**). *P < 0.05, **,*p* < 0.01, ***p < 0.001 compared with control

Bioinformatics analysis using the TargetScan, miRWALK, and miRBASE databases jointly predicted the presence of a highly conserved binding site between miR-224-5p and the 3’-UTR of the PTEN gene (Fig. 5A). A dual-luciferase reporter assay demonstrated that miR-224-5p significantly suppressed the activity of the PTEN 3’-UTR (Fig. 5B), thereby confirming their direct regulatory relationship at the molecular interaction level.

The results of the Western blot analysis revealed that compared with the control treatment, the overexpression of miR-224-5p led to a significant downregulation of PTEN protein expression (Fig. 5C–E).

### Rescue experiments: interaction between miR-224-5p and the PTEN/PI3K/AKT signaling pathway

**Fig. 6:**
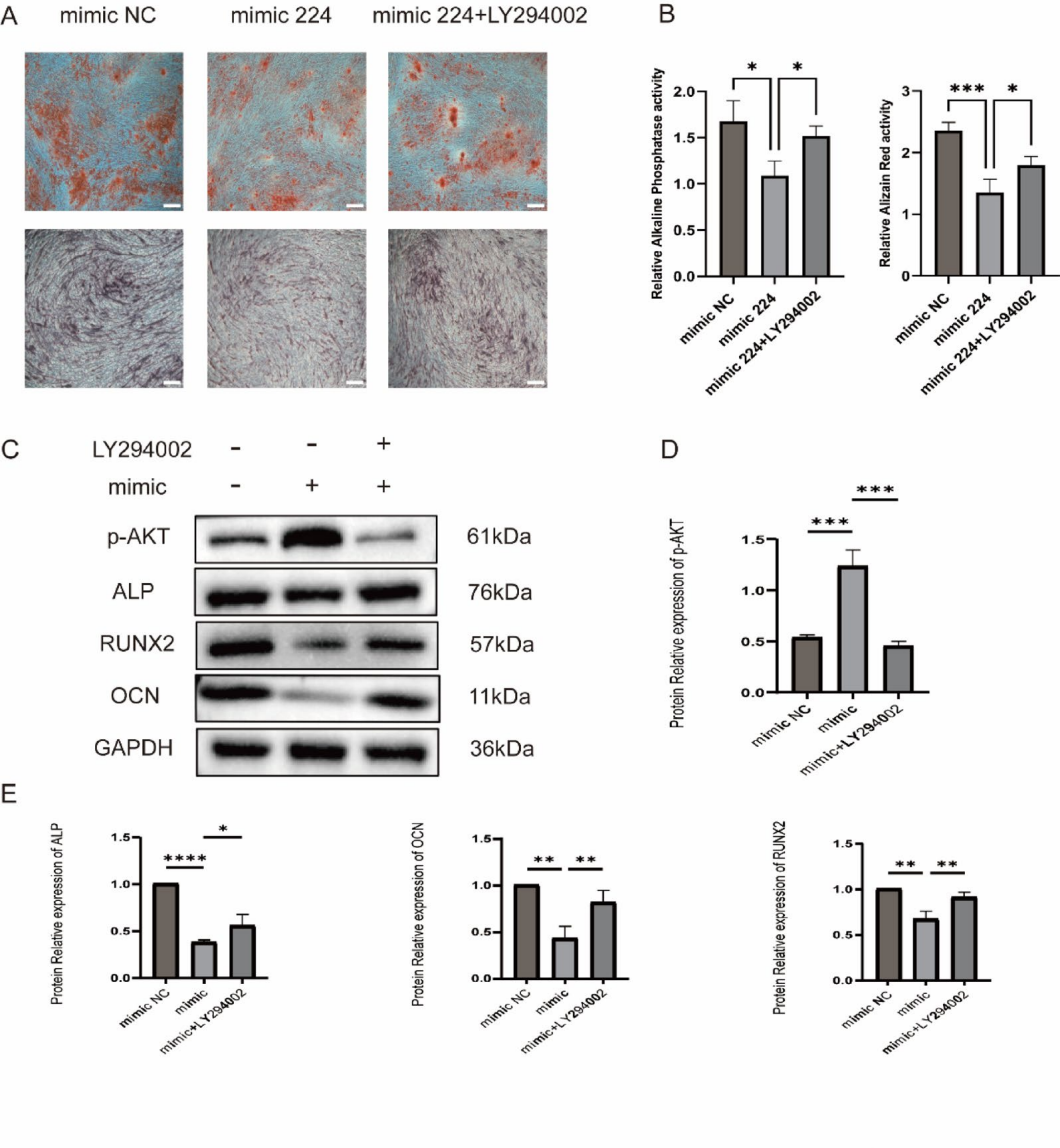
ALP activity and Alizarin Red Staining in the miR-224-5p overexpression group and the miR-224-5p overexpression combined with LY294002 group(A). Scale bar, 10×, 100 μm. The results of the Western blot analysis of p-AKT, ALP, RUNX2, and OCN protein expression levels after transfection of miR-224-5p mimic plus LY294002(C). The results of the quantitative analysis are shown in Fig. 6B, D and E. * *P* < 0.05, ** *p* < 0.01, *** *p* < 0.001, **** *p* < 0.0001 compared with the control.

Following osteogenic induction of human dental pulp stem cells (hDPSCs), alizarin red S staining and alkaline phosphatase (ALP) activity assay were performed (Fig. 6A). Quantitative analysis showed that both alizarin red S staining and ALP activity were significantly decreased in the miR‑224‑5p upregulated group compared with the blank control group. In contrast, these indicators were markedly elevated in the group co‑treated with miR‑224‑5p mimic and LY294002, relative to the miR‑224‑5p mimic‑treated group (Fig. 6B). Compared with the group co-treated with miR-224-5p mimics and LY294002, the miR-224-5p overexpression group exhibited lower calcium nodule formation rate and ALP activity level, as well as decreased protein expression levels of related osteogenic markers (Runx2, ALP, OCN).

The results demonstrated that addition of the miR-224-5p mimic significantly increased AKT phosphorylation. Western blot analysis revealed that the expression levels of osteogenic marker proteins, including Runx2, ALP, and OCN, were significantly downregulated in the miR‑224‑5p upregulated group compared with the blank control group. Conversely, co‑treatment with miR‑224‑5p mimic and LY294002 significantly upregulated the expression of these proteins relative to treatment with miR‑224‑5p mimic alone (Fig. 6C–E).

## Discussion

Stem cells from the apical papilla (SCAPs) exhibit robust osteogenic differentiation potential, yet their isolation and acquisition remain technically challenging. In our previous study, we identified that miR-224-5p was the most significantly differentially expressed microRNA in the differential expression profiles between SCAPs and DPSCs [18]. Based on this finding, we hypothesized that miR-224-5p may play a pivotal role in the osteogenic process. Therefore, the aim of this study was to investigate the underlying mechanism by which miR-224-5p modulates osteoblastic differentiation. Our experimental results demonstrated that miR-224-5p could directly target and repress PTEN expression, thereby regulating the osteogenic differentiation of DPSCs through the PTEN/PI3K/AKT signaling pathway.

In this study, we isolated and sorted dental pulp cells from dental pulp tissue and selected STRO-1(+) cells for further experiments [27]. These cells have osteogenic and adipogenic differentiation potential, and detection of surface marker expression revealed that the cells we isolated were negative for CD34 and CD45 expression and positive for that of CD90 and CD73. These results are in accordance with the standards for the identification of mesenchymal stem cells [26].

miRNAs were first discovered by Ambros and Ruvkun in 1993 [28]. It is now known that the human genome encodes more than a thousand miRNAs, which play crucial roles in the normal development of cells and tissues. MiRNAs accelerate tendon healing through the modulation of tenocyte proliferation, differentiation and angiogenesis [29], and are also implicated in the pathophysiological processes of osteoarthritis by regulating chondrocyte proliferation, apoptosis and inflammatory responses, as well as extracellular matrix (ECM) metabolism [30, 31].Abnormal or mutated miRNAs can lead to various diseases, including cancer, congenital hearing loss [32], eye disorders [33, 34], and osteoporosis [35, 36]. Researchers have also taken advantage of these characteristics to develop treatment plans to treat these diseases. In recent years, researchers have explored the relationship between the genome and miRNAs. The genome shapes the expression dynamics of miRNAs through cell type-specific transcriptional regulation; the origins, distributions, and functions of miRNAs, in turn, interact with the genome [37–39].The experimental results clearly reveal that upregulating the expression of miR-224-5p inhibits the osteogenic differentiation of hDPSCs and that inhibiting the expression of miR-224-5p promotes the osteogenic differentiation of hDPSCs. The specific manifestations are an increase in calcified nodule numbers and an increase in ALP activity. Previous studies have shown that miR-224-5p has a negative regulatory effect on osteoblast differentiation [40]. These results are in line with those of our study.

Through gene prediction, we determined that PTEN is one of the functional target genes of miR-224-5p. We conducted a series of experiments, including bioinformatics prediction, validation of molecular interactions, and protein-level detection, and multidimensionally confirmed that PTEN is a direct target gene of miR-224-5p. These findings not only reveal the existence of a miR-224-5p-PTEN regulatory axis but also provide important clues for understanding the molecular mechanism of miR-224-5p in osteogenic differentiation. MiR-374b has been demonstrated to degrade PTEN, thereby promoting osteogenic differentiation of mesenchymal stem cells (MSCs) and accelerating fracture healing [41]. In parallel, miR-29b targets PTEN to facilitate the osteogenic differentiation of human adipose-derived mesenchymal stem cells (hADMSCs) [42]. MiR-106a-5p promotes the proliferation and inhibits the apoptosis of osteoblasts (hFOB1.19) by targeting and inhibiting PTEN [43].

Most of the known functions of PTEN are achieved through regulation of the PI3K/AKT pathway [44]. Studies have revealed that miR-374-5p suppresses osteogenic differentiation in primary rat osteoblasts by modulating the PTEN/PI3K/AKT signaling pathway [41]. MiR-142-5p promotes osteoclast differentiation in bone marrow-derived macrophages (BMMs) via the PTEN/PI3K/AKT/FoxO1 pathway [45]. Additionally, miR-363-3p targets PTEN to activate the PI3K/AKT signaling pathway, thereby negatively regulating the expression levels of osteoblastic marker genes, including OC, ALP and COL1A1 [46]. Furthermore, exosomal miR-501-3p derived from osteosarcoma cells activates the PI3K/Akt/NFATc1 pathway by repressing PTEN expression in bone marrow-derived macrophages (BMDMs), ultimately facilitating osteoclast differentiation [47]. However, its interaction with miR-224-5p during osteogenic differentiation of hDPSCs remains inadequately explored. In this study, LY294002 was used to inhibit the activity of PI3K. Moreover, overexpression of miR-224-5p in hDPSCs inhibited osteogenic differentiation, whereas inhibition of the PI3K/AKT pathway reversed the biological effects of miR-224-5p overexpression on hDPSCs. These results confirm that miR-224-5p mediates osteogenic differentiation in hDPSCs to a certain extent by regulating PI3K/AKT.

This study clearly illustrates that miR-224-5p may regulate the osteogenic differentiation of human dental pulp stem cells by modulating the PTEN/PI3K/AKT signaling pathway. Although we demonstrate that the modulation of the PTEN/PI3K/AKT signaling pathway exerts a prominent effect on metabolism and osteogenesis in our experimental settings, other signaling pathways are likely to be involved, which warrants further experimental validation. In future research, in vivo studies may be performed to obtain systemic results, thereby providing experimental data support for the clinical repair of bone defects.

## Conclusions

MiR-224-5p can target and regulate osteogenic differentiation in dental pulp stem cells through the PTEN/PI3K/AKT pathway. Specifically, inhibiting the expression of miR-224-5p can promote osteogenic differentiation of dental pulp stem cells. This is highly important for clinical applications related to synthesizing new bone and promoting tissue healing.

## Supplementary Information

Below is the link to the electronic supplementary material.

Supplementary Material 1

## Data Availability

The datasets generated and/or analyzed during this study are available from the corresponding author upon reasonable request.

## Abbreviations

hDPSCs: Human dental pulp stem cells
RUNX2: Runt-related transcription factor 2
ALP: Alkaline phosphatase
OCN: Osteocalcin
MSCs: Mesenchymal stem cells
SCAPs: Stem cells from the apical papilla
miRNAs: MicroRNAs
PBS: Phosphate-buffered saline
FBS: Fetal bovine serum
DMEM: Dulbecco’s modified Eagle’s medium
FACS: Flow cytometry staining buffer
cDNA: Complementary DNA
ARS: Alizarin red S
PMSF: Phenylmethylsulfonyl fluoride
SDS: Sodium dodecyl sulfate
PVDF: Polyvinylidene fluoride
BSA: Bovine serum albumin
HRP: Horseradish peroxidase
RT-qPCR: Quantitative real-time polymerase chain reaction
GAPDH: Glyceraldehyde-3-phosphate dehydrogenase
