# Supplementary material for: Inhibition of miR-224-5p promotes osteogenesis in dental pulp stem cells by targeting the PTEN/PI3K/AKT axis
Source: J Orthop Surg Res. 2026 Apr 11;21:335. doi: 10.1186/s13018-026-06853-w (PMC13270850; doi:10.1186/s13018-026-06853-w)
Supplement: Supplementary file 1 — Supplementary Material 1 [file 13018_2026_6853_MOESM1_ESM.pdf]

Osteogenic Differentiation

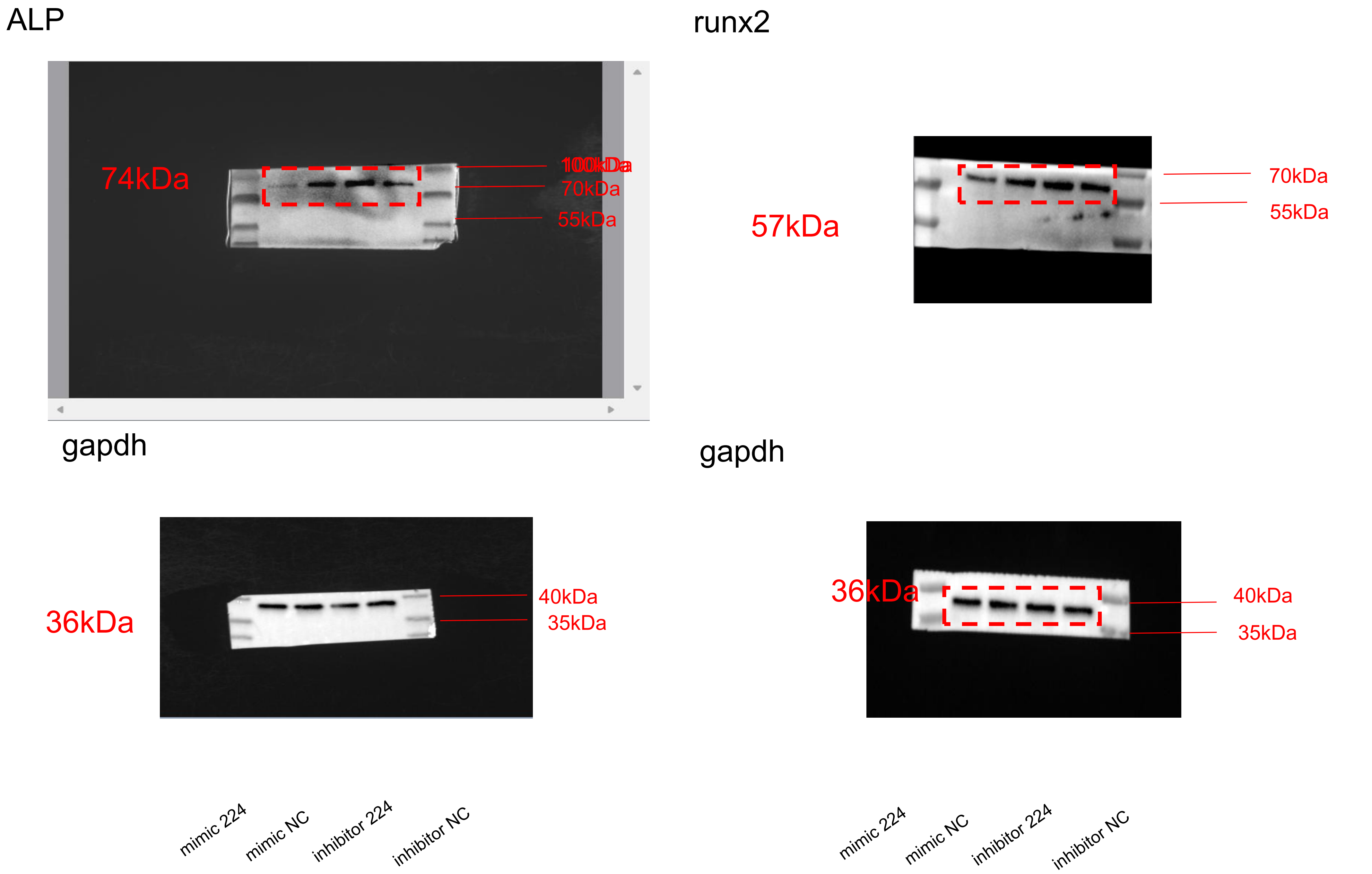

Osteogenic Differentiation

ocn

11kDa

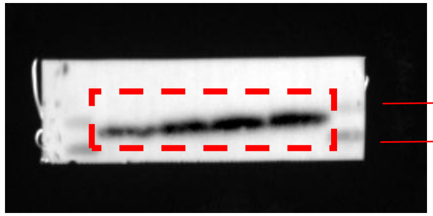

gapdh

36kDa

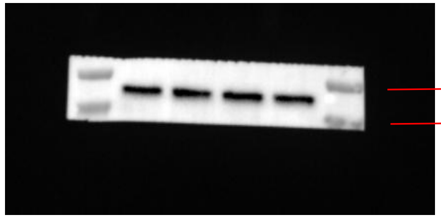

mimic 224
mimic NC
inhibitor 224
inhibitor NC

PTEN mimic

PTEN inhibitor

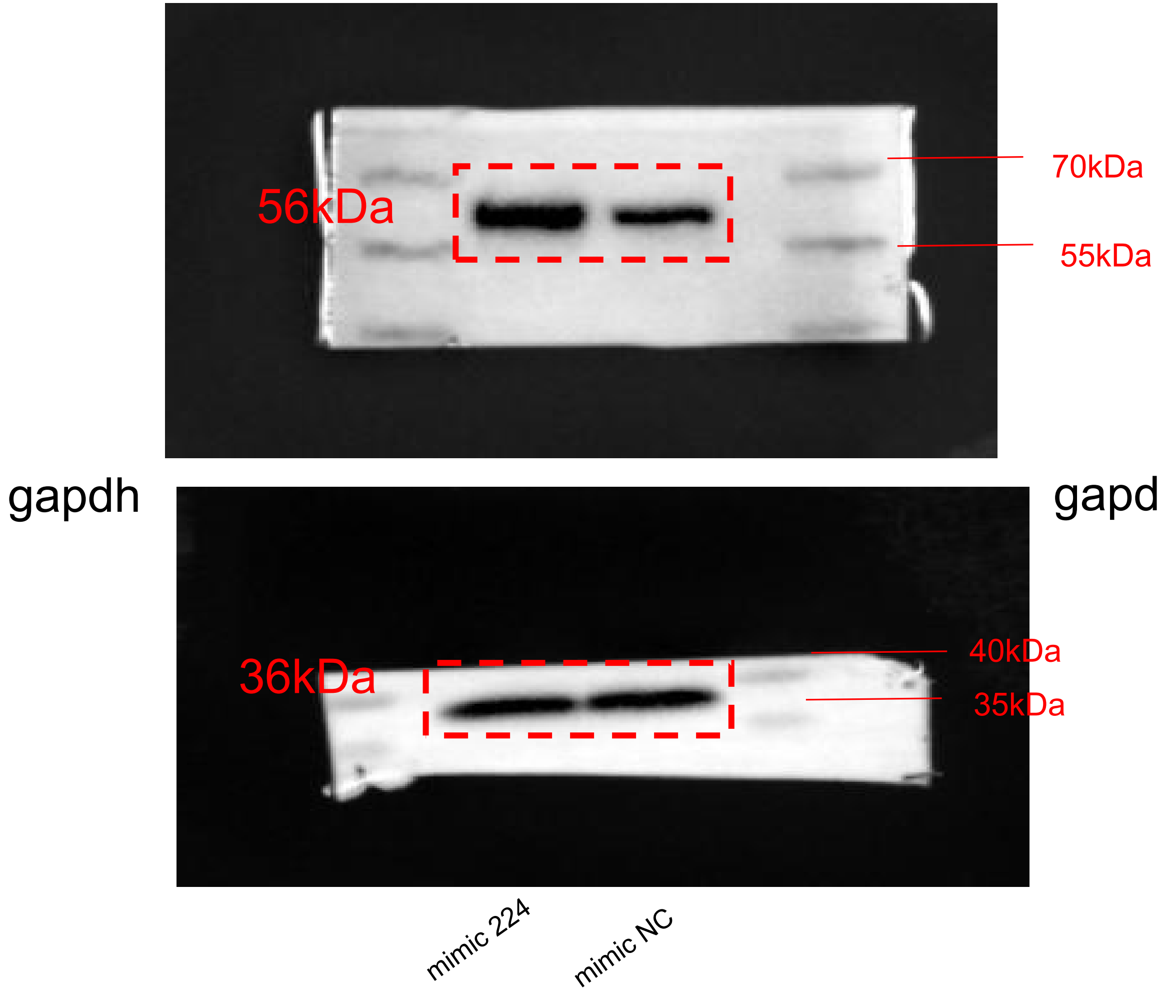

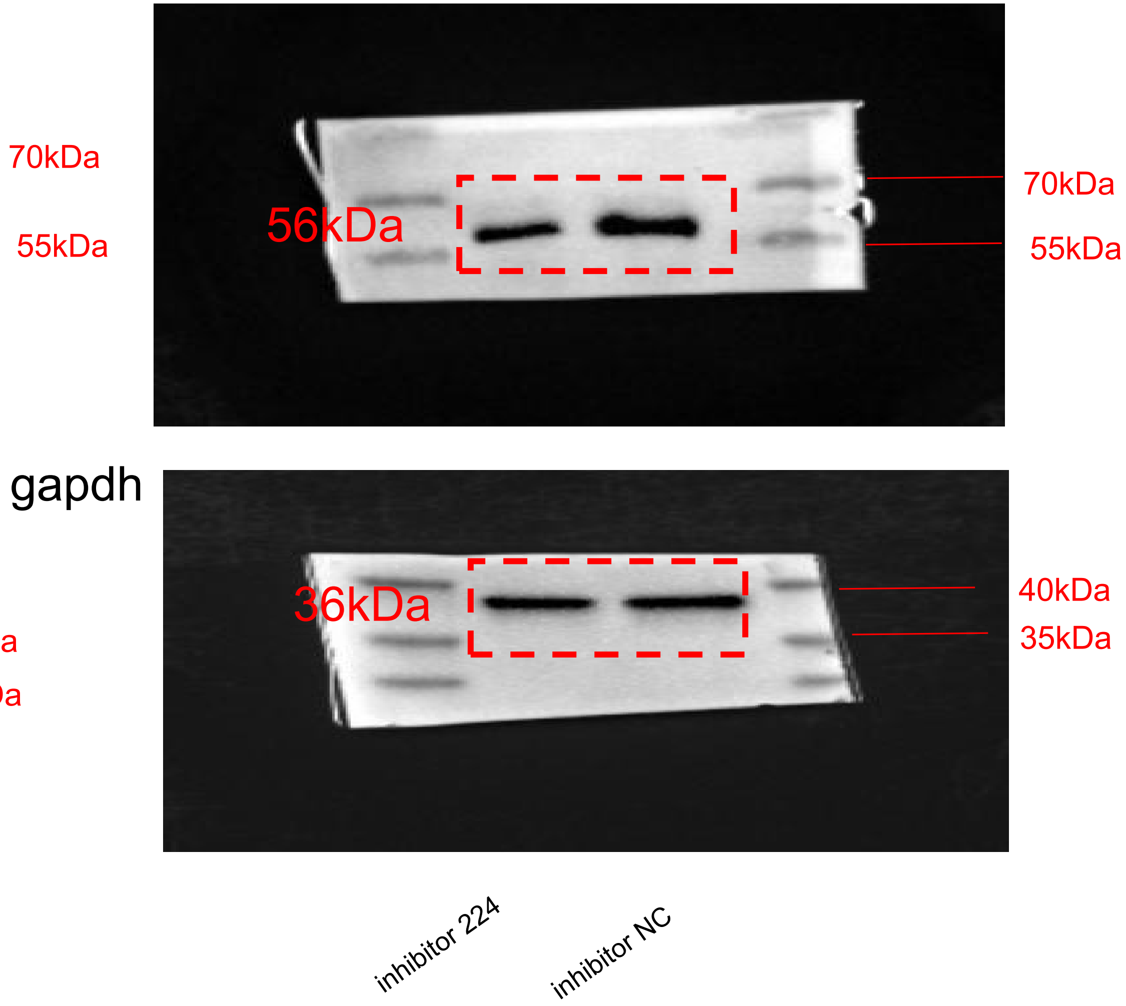

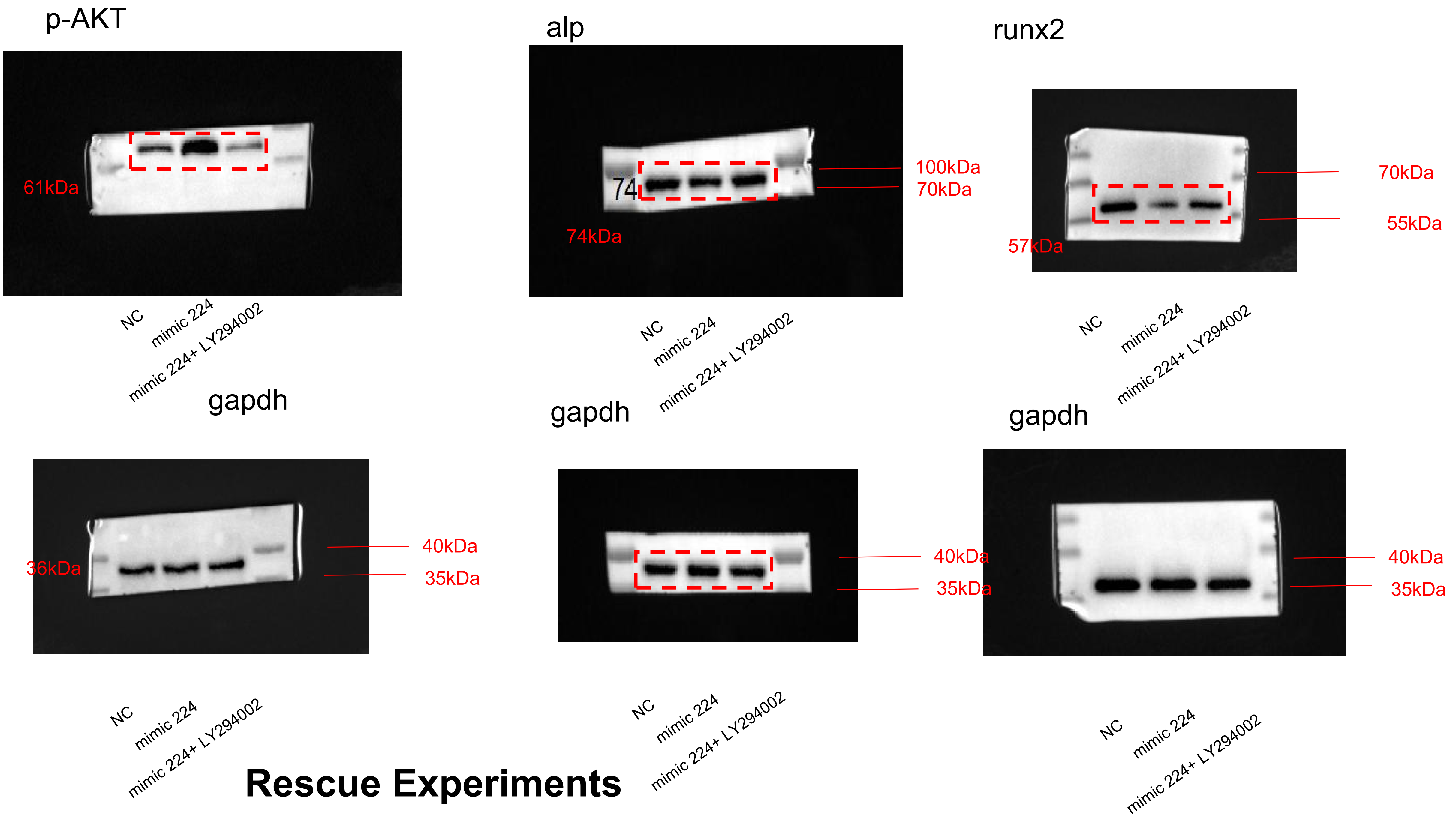

Rescue Experiments

ocn

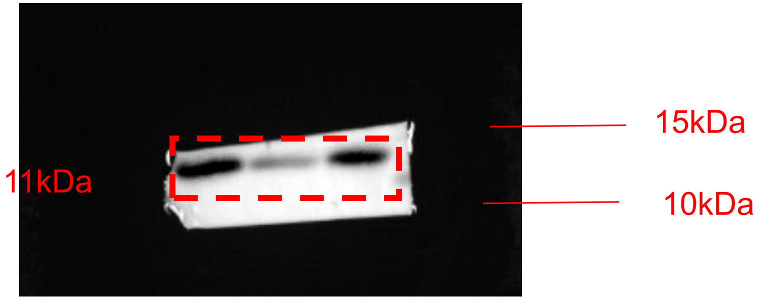

gapdh

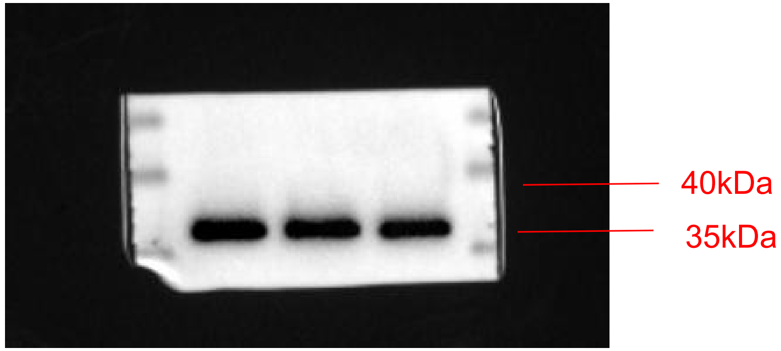

Rescue Experiments
